# Supplementary material for: Dissipative optomechanics in high-frequency nanomechanical resonators
Source: Nat Commun. 2023 Sep 18;14:5793. doi: 10.1038/s41467-023-41127-7 (PMC10507050; doi:10.1038/s41467-023-41127-7)
Supplement: Supplementary file 2 — Supplementary Information [file 41467_2023_41127_MOESM2_ESM.pdf]

Supplemental Material: Dissipative Optomechanics in High-Frequency Nanomechanical Resonators

André G. Primo,[1,*] Pedro V. Pinho,[1,*] Rodrigo Benevides,[2] Simon Gröblacher,[3] Gustavo S. Wiederhecker,[1] and Thiago P. Mayer Alegre[1,†]

[1] *Gleb Wataghin Institute of Physics, University of Campinas, 13083-859 Campinas, SP, Brazil*
[2] *Department of Physics, ETH Zürich, 8093 Zürich, Switzerland*
[3] *Kavli Institute of Nanoscience, Department of Quantum Nanoscience, Delft University of Technology, Lorentzweg 1, 2628CJ Delft, The Netherlands*

(Dated: September 7, 2023)

S1. FROM AN EIGENVALUE PROBLEM TO DISSIPATIVE OPTOMECHANICS

Here we aim at deriving the existence of a dissipative optomechanical coupling using the system of coupled cavities in Fig.**1b** of the main text. We generalize the discussion by allowing both "bare" optical modes to display different frequencies ω_1 and $\omega_2 = \omega_1 - \Delta\omega$. The amplitude of the optical fields a_1 and a_2 are described as[1]:

$$\frac{d}{dt}\begin{bmatrix} a_1 \\ a_2 \end{bmatrix} = \begin{bmatrix} -i\left(\omega_1 - G_\omega x\right) - \frac{(\kappa_e+\kappa_i)}{2} & iJ \\ iJ & -i\left(\omega_1 - \Delta\omega\right) - \frac{\kappa_i}{2} \end{bmatrix} \cdot \begin{bmatrix} a_1 \\ a_2 \end{bmatrix} - \begin{bmatrix} \sqrt{\kappa_e}\bar{\alpha}_{\text{in}} e^{-i\omega_l t} \\ 0 \end{bmatrix}, \tag{S1}$$

which can be written in the form of $\frac{d}{dt}\vec{a} = \mathbf{M}\cdot\vec{a} + \mathbf{J}$ and $\bar{\alpha}_{\text{in}}$ is a coherent driving amplitude.

In the experiment, we probe this system through its supermode response, or the eigenvectors of the matrix $\mathbf{M}$. The eigenvalues of $\mathbf{M}$, $E_\pm$, are given by:

$$E_\mp = -i\left(\omega_1 - \frac{\Delta\omega}{2} - \frac{G_\omega}{2}x\right) - \frac{\kappa_i}{2} - \frac{\kappa_e}{4} \pm i\sqrt{J^2 + \left[\frac{2(-\Delta\omega + G_\omega x) + i\kappa_e}{4}\right]^2}, \tag{S2}$$

which can be Taylor expanded keeping terms only to the first order in x, yielding:

$$E_\mp = -i\left(\omega_1 - \frac{\Delta\omega}{2}\right) - \frac{\kappa_i}{2} - \frac{\kappa_e}{4} \pm i\sqrt{J^2 + \left(\frac{-2\Delta\omega + i\kappa_e}{4}\right)^2} + \left(i\frac{G_\omega}{2} \mp \frac{G_\omega}{2}\frac{i2\Delta\omega + \kappa_e}{4\sqrt{J^2 + \left(\frac{2\Delta\omega - i\kappa_e}{4}\right)^2}}\right)x. \tag{S3}$$

The terms independent of x define the unperturbed response of the coupled resonators. Their imaginary (real) parts correspond to the frequencies (loss rates) of the supermodes. In the notation adopted in the main text, E_+ is associated with ω_+, κ_+ and E_- with ω_-, κ_-. In the regime $J \gg \kappa_e$, corresponding to our experiment, the eigenfrequencies split into two branches. This is shown in Fig. 1**c** of the main text, as a function of $\Delta\omega$. Naturally, if $|\Delta\omega| \gg J$ we should recover the uncoupled cavity limit. This is mathematically equivalent to taking $J \to 0$ in the equations above. Importantly, our device operates in a regime $\Delta\omega/J \ll 1$, which is near optimal. In fact, for $\Delta\omega = 0$ we note that the losses of both supermodes are identical and their frequencies differ by a factor $2\sqrt{J^2 - \kappa_e^2/16} \approx 2J$, indicating a perfect optical hybridization, that is, $\omega_\pm \approx \omega_1 \pm J$ and $\kappa_\pm = \kappa_i + \frac{\kappa_e}{2}$. From now on, we assume $\Delta\omega \to 0$.

The linear terms on x generate the optomechanical coupling. For simplicity, we may consider the terms multiplying x as an effective optomechanical coupling. We have

$$\lim_{\Delta\omega\to 0}\left(i\frac{G_\omega}{2} \mp \frac{G_\omega}{2}\frac{i2\Delta\omega + \kappa_e}{4\sqrt{J^2 + \left(\frac{2\Delta\omega - i\kappa_e}{4}\right)^2}}\right)x = \left(i\frac{G_\omega}{2} \mp \frac{G_\omega}{2}\frac{\kappa_e}{4\sqrt{J^2 - \left(\frac{\kappa_e}{4}\right)^2}}\right)x, \tag{S4}$$

* agprimo@ifi.unicamp.br, ppinho@ifi.unicamp.br; These authors contributed equally to this work.
† alegre@unicamp.br

from which we see that the effective optomechanical coupling has both an imaginary and a real part, which are associated with dispersive and dissipative couplings, respectively [2]. Here, the dispersive component is divided by a factor of 2 when compared to the uncoupled cavity regime, and is identical for both supermodes, i.e. $G_{\omega\pm} = \frac{G_\omega}{2}$. The effective dissipative coupling is given by

$$G_{\kappa_{e\pm}} = \mp G_\omega \frac{\kappa_e}{4\sqrt{J^2 - (\frac{\kappa_e}{4})^2}}, \tag{S5}$$

and associated with a modulation in the extrinsic losses, since this is the dominant loss asymmetry in our analysis. In our experiment, $g_\omega/(2\pi) \approx 700\,\text{kHz}$, $\kappa_e/(2\pi) \approx 280\,\text{MHz}$, and $J/(2\pi) \approx 15\,\text{GHz}$, where g_ω and κ_e were obtained by summing the measured values of $g_{\omega\pm}$ and $\kappa_{e\pm}$. This is accurate since the eigenvalue treatment of the problem points out that the bare cavity dispersive coupling and extrinsic loss are, in fact, split between the two supermodes (if $\Delta\omega \neq 0$ this splitting is uneven). Plugging the experimental values in Eq. S5, we find $g_{\kappa_{e\pm}}/(2\pi) \approx \mp 3.26\,\text{kHz}$, in excellent agreement with our measurements.

The source term in Eq. S1 is treated as follows: the eigenvectors of $\mathbf{M}$, $a_\pm$, are related to a_1 and a_2 through:

$$\begin{bmatrix} a_+ \\ a_- \end{bmatrix} = \mathbf{P}(x) \cdot \begin{bmatrix} a_1 \\ a_2 \end{bmatrix}, \tag{S6}$$

where $\mathbf{P}(x)$ is the basis transformation matrix. If $\Omega \ll 2J$, i.e. the adiabatic regime, we may assume that the time variations of $\mathbf{P}(x)$ are slow compared to those of a_1 and a_2. This can be readily seen by moving into a frame rotating at the laser frequency ω_l. In this case, we can rewrite Eq. S1 in terms of the column vector $\vec{a}_S = [a_+, a_-]^t$ as:

$$\frac{d}{dt}\vec{a}_S = \mathbf{P} \cdot \mathbf{M} \cdot \mathbf{P}^{-1} \cdot \vec{a}_S + \mathbf{P} \cdot \mathbf{J}, \tag{S7}$$

meaning the driving term is now dependent on x. The matrix $\mathbf{P} \cdot \mathbf{M} \cdot \mathbf{P}^{-1}$ is diagonal with the eigenvalues $E_\pm$ as its non-zero terms. The matrix $\mathbf{P}$ is given by:

$$\mathbf{P}(x) = \frac{1}{\sqrt{2}} \begin{bmatrix} -1 - \frac{G_{\kappa_{e+}}}{\kappa_e} x & 1 - \frac{G_{\kappa_{e+}}}{\kappa_e} x \\ 1 + \frac{G_{\kappa_{e-}}}{\kappa_e} x & 1 - \frac{G_{\kappa_{e-}}}{\kappa_e} x \end{bmatrix}, \tag{S8}$$

where we also used $\kappa_e \ll J$ and neglected nonlinear terms in x. Finally, defining $\kappa_{e\pm} = \frac{\kappa_e}{2}$ we have:

$$\frac{d}{dt}\begin{bmatrix} a_+ \\ a_- \end{bmatrix} = \begin{bmatrix} -i\left(\omega_+ - G_{\omega_+}x\right) - \frac{(\kappa_+ + G_{\kappa_{e+}}x)}{2} & 0 \\ 0 & -i\left(\omega_- - G_{\omega_-}x\right) - \frac{(\kappa_- + G_{\kappa_{e-}}x)}{2} \end{bmatrix} \cdot \begin{bmatrix} a_+ \\ a_- \end{bmatrix} - \begin{bmatrix} -\sqrt{\kappa_{e+}} - \frac{G_{\kappa_{e+}}}{2\sqrt{\kappa_{e+}}}x \\ \sqrt{\kappa_{e-}} + \frac{G_{\kappa_{e-}}}{2\sqrt{\kappa_{e-}}}x \end{bmatrix} \bar{\alpha}_{\text{in}} e^{-i\omega_l t}. \tag{S9}$$

S2. GENERALIZED OPTOMECHANICAL TRANSDUCTION

We turn our attention to the description of the fluctuations imprinted in the optical field due to the thermo-mechanical noise. Since our supermodes are several mechanical frequencies away from each other, we can analyze their response individually. We start from the classical optical field description:

$$\dot{a} = i(\Delta + G_\omega x)a - \frac{\kappa + G_{\kappa_e}x}{2}a - \sqrt{\kappa_e}\bar{\alpha}_{\text{in}} - \frac{G_{\kappa_e}x}{2\sqrt{\kappa_e}}\bar{\alpha}_{\text{in}}, \tag{S10}$$

where we moved into a reference frame rotating at the laser frequency ω_l, therefore, $\Delta = \omega_l - \omega_c$, where ω_c is the mode's frequency. Here, we can associate a, Δ, κ, and κ_e with either of the supermodes. We can linearize this equation around stationary coherent amplitudes $\bar{x}$ and $\bar{a}$ by making $a(t) = \bar{a} + \delta a(t)$ and $x(t) = \bar{x} + \delta x(t)$, and keeping terms only to first order in the fluctuations. The coherent electromagnetic field is given by:

$$\bar{a} = \frac{\sqrt{\bar{\kappa}_e}\bar{\alpha}_{\rm in}}{i\bar{\Delta} - \frac{\bar{\kappa}}{2}}, \tag{S11}$$

and the dynamical part reads:

$$\dot{\delta a} = i(\bar{\Delta}\delta a + G_\omega \bar{a}\delta x) - \frac{\bar{\kappa}\delta a + G_{\kappa_e}\bar{a}\delta x}{2} - \frac{G_{\kappa_e}\delta x}{2\sqrt{\bar{\kappa}_e}}\bar{\alpha}_{\rm in}, \tag{S12}$$

where $\bar{\Delta}$, $\bar{\kappa}$, $\bar{\kappa}_e$ are the renormalized detuning, total and extrinsic losses, respectively, accounting for the effects arising from a static shift in the mechanical displacement, $\bar{x}$.

The form of Eq. S12 allows a clear interpretation of the several sources contributing to the optical field fluctuations. First, we have the dispersively scattered part of δa, which is seeded by the pump photons $\bar{a}$. On the dissipative side, we have source terms proportional to $\bar{a}$ and to $\bar{\alpha}_{\rm in}$. The first is enhanced by the coherent part of the electromagnetic field, in the same fashion as the dispersive coupling contribution, whereas the latter is related to the waveguide-cavity scattering mechanism and is central to our discussion. In fact, for largely off-resonant excitation, which is the relevant case in sideband-resolved optomechanical systems, we have $\bar{a} \approx \frac{\sqrt{\bar{\kappa}_e}\bar{\alpha}_{\rm in}}{i\bar{\Delta}}$. This is the enhancement factor for dispersive optomechanical coupling. In the case of waveguide-cavity scattering, the expression playing this same role is $\frac{\bar{\alpha}_{\rm in}}{2\sqrt{\bar{\kappa}_e}}$, which is a factor $\approx \Delta/(2\kappa_e)$ larger than its dispersive counterpart. In our experiment, $\Delta/(2\kappa_e) \approx 20$, justifying the large contribution from dissipative optomechanics at large detunings. Near the resonance, however, this term decreases, and so does the dissipative contribution.

The photocurrent generated at our detector is a function of the field reflected by our resonator. This is obtained through input-output relations:

$$\alpha_{\rm out}(t) = \bar{\alpha}_{\rm in} + \sqrt{\bar{\kappa}_e}\left(\bar{a} + \delta a(t)\right) + \frac{G_{\kappa_e}\bar{a}}{2\sqrt{\bar{\kappa}_e}}\delta x(t). \tag{S13}$$

The output field $\alpha_{\rm out}(t)$ can also be linearized as $\alpha_{\rm out}(t) = \bar{\alpha}_{\rm out} + \delta\alpha_{\rm out}(t)$, and fluctuating terms can be matched between the right-hand and left-hand sides of Eq. S13. The photocurrent $I(t)$ is proportional to $|\alpha_{\rm out}(t)|^2$, whose fluctuations are given by $\delta I(t) = \bar{\alpha}_{\rm out}\delta\alpha^*_{\rm out}(t) + \bar{\alpha}^*_{\rm out}\delta\alpha_{\rm out}(t)$. Using a spectrum analyzer, one measures the power spectral density of $\delta I(t)$, $S_{II}(\omega) = \int d\tau e^{i\omega\tau}\langle\delta I(\tau)\delta I(0)\rangle$. Finding this quantity requires moving into a frequency domain description in Eq. S12, where the optical field $\delta a(\omega)$ can be written solely in terms of $\delta x(\omega)$. We find

$$S_{II}(\omega) = \frac{16|\bar{\alpha}_{\rm in}|^4|\left(-\bar{\kappa}_e + \bar{\kappa} - i\omega\right)\left(4\bar{\Delta}^2 G_{\kappa_e} - 8\bar{\Delta}\bar{\kappa}_e G_\omega + \left(\bar{\kappa} - 2\bar{\kappa}_e\right)\left(\bar{\kappa} - 2i\omega\right)G_{\kappa_e}\right)|^2}{\left(4\bar{\Delta}^2 + \bar{\kappa}^2\right)^2\left(8\bar{\Delta}^2\left(\bar{\kappa}^2 - 4\omega^2\right) + 16\bar{\Delta}^4 + \left(\bar{\kappa}^2 + 4\omega^2\right)^2\right)} S_{xx}(\omega), \tag{S14}$$

where S_{xx} is the mechanical power spectral density. Assuming a weak optomechanical coupling, S_{xx} is related to the bare mechanical response, in the absence of optomechanical backaction, and therefore is independent of $\bar{\Delta}$. As a consequence, the prefactor of S_{xx} in Eq. S14 can be used as a model for optomechanical transduction. Here, we omitted a prefactor related to the particularities of the detection system used, such as signal losses, photodetector gain, and efficiency.

S3. DYNAMICAL BACKACTION IN A DISSIPATIVE OPTOMECHANICAL SYSTEM

The Hamiltonian of a generalized optomechanical system in the presence of both dissipative and dispersive couplings is given by: $\hat{H} = \hbar\omega_c\hat{a}^\dagger\hat{a} + \hbar\Omega\hat{b}^\dagger\hat{b} + \sum_q \hbar\omega_{q,i}\hat{c}^\dagger_{q,i}\hat{c}_{q,i} + \sum_q \hbar\omega_{q,e}\hat{c}^\dagger_{q,e}\hat{c}_{q,e} + \hat{H}_\kappa + \hat{H}_\Gamma + \hat{H}_{\rm OM}$. The first two terms in $\hat{H}$ describe the bare optical and mechanical oscillators. $\hat{H}_\kappa$ and $\hat{H}_\Gamma$ describe the (unperturbed) damping of the optics (κ) and mechanics (Γ), respectively, which carry no dependency on $\hat{x}$. In particular, optical losses have both intrinsic and extrinsic contributions, related to the third and fourth terms describing the respective bosonic baths with subscripts "i" and "e". Assuming dispersive and extrinsic dissipative optomechanical interactions, the interaction Hamiltonian $\hat{H}_{\rm OM}$ has the form:

$$\hat{H}_{\rm OM} = -\left[\hbar G_\omega\hat{a}^\dagger\hat{a} + i\sqrt{\frac{\kappa_e}{2\pi\rho_e}}\frac{\hbar G_{\kappa_e}}{2\kappa_e}\sum_q\left(\hat{a}^\dagger\hat{c}_{q,e} - \hat{c}^\dagger_{q,e}\hat{a}\right)\right]\hat{x}. \tag{S15}$$

Here, $\hat{x}$ is the mechanical position operator and ρ_e denotes the density of states of the waveguide. In the Markovian limit ($\omega_c \gg \kappa$), we may treat ρ_e as a constant. We assume only the coupling to the extrinsic bath modes to be affected by the mechanical displacement, ignoring the so-called intrinsic dissipative coupling. Tracing out the optical baths by means of the input-output formalism, the linearized optical force operator is $\hat{F} = -\frac{\partial \hat{H}_{\mathrm{OM}}}{\partial \hat{x}}$ [3, 4]

$$\begin{aligned}\hat{F} x_{\mathrm{zpf}} &= \hbar g_\omega \left(\bar{a}^* \delta\hat{a} + \bar{a}\delta\hat{a}^\dagger\right) \\ &\quad + i\frac{\hbar g_{\kappa_e}}{2\kappa_e}\sqrt{\kappa_{\mathrm{e}}}\left[\bar{a}^*\delta\hat{\alpha}_{\mathrm{in}}^{\mathrm{e}} - \left(\delta\hat{\alpha}_{\mathrm{in}}^{\mathrm{e}}\right)^\dagger \bar{a}\right] + i\frac{\hbar g_{\kappa_e}}{2\kappa_e}\sqrt{\kappa_e}\left[\bar{\alpha}_{\mathrm{in}}\delta\hat{a}^\dagger - \bar{\alpha}_{\mathrm{in}}^*\delta\hat{a}\right].\end{aligned} \tag{S16}$$

Here, we linearized the optical dynamics around coherent ($\bar{a} = \langle \hat{a}\rangle$) and fluctuation ($\delta\hat{a}$) amplitudes, in the form of $\hat{a} = \bar{a} + \delta\hat{a}$. The dissipation-fluctuation theorem [5] shows that the coupling to the bath modes will effectively add noise to the optical field (modeled as a fluctuating source term). In our system, this noise is intrinsic ($\delta\hat{\alpha}_{\mathrm{in}}^{\mathrm{i}}$) or extrinsic ($\delta\hat{\alpha}_{\mathrm{in}}^{\mathrm{e}}$) in nature. Since the coupling to the extrinsic bath is modulated by the mechanics, $\delta\hat{\alpha}_{\mathrm{in}}^{e}$ also appears in the force operator.

The first line in Eq. S16 describes the force arising from the dispersive coupling, while the second is related to the extrinsic dissipative coupling. The last term is particularly interesting since it gives rise to a force directly dependent on the coherent drive amplitude through the extrinsic channel. For telecom optical photons, we may assume a zero temperature optical bath, with noise correlations: $\left\langle \delta\hat{\alpha}_{\mathrm{in}}^{J}(\omega)(\delta\hat{\alpha}_{\mathrm{in}}^{J'}(\omega'))^\dagger\right\rangle = 2\pi\delta\left(\omega+\omega'\right)\delta_{J,J'}$, $J = \mathrm{e}, \mathrm{i}$, meaning the intrinsic and extrinsic noises are uncorrelated. In the weak coupling regime ($C \ll \kappa/\Gamma$), one may compute the backaction cooling/amplification of the mechanical mode from the unperturbed force's noise spectrum $S_{\mathrm{FF}}(\Omega)$[3, 4, 6] and the Fermi's Golden Rule. In the present case, we have $\delta\Gamma = \frac{x_{\mathrm{zpf}}^2}{\hbar^2}\left[S_{\mathrm{FF}}(\Omega) - S_{\mathrm{FF}}(-\Omega)\right]$. Plugging in the expression for S_{FF}, we have

$$\delta\Gamma = -\frac{4n_c\Omega}{\bar{\kappa}_e^2}\frac{\left\{2g_\omega g_{\kappa_e}\bar{\kappa}_e\left[\bar{\kappa}_e\left(-4\bar{\Delta}^2+\bar{\kappa}^2+4\Omega^2\right)-8\bar{\Delta}^2\bar{\kappa}\right]+\bar{\Delta}g_{\kappa_e}^2\left[4\bar{\Delta}^2\bar{\kappa}+\bar{\kappa}_e\left(4\bar{\Delta}^2-3\bar{\kappa}^2-4\Omega^2\right)+\bar{\kappa}^3\right]+16\bar{\Delta}\bar{\kappa}_e^2\bar{\kappa}g_\omega^2\right\}}{16\bar{\Delta}^4+8\bar{\Delta}^2\left(\bar{\kappa}^2-4\Omega^2\right)+\left(\bar{\kappa}^2+4\Omega^2\right)^2}, \tag{S17}$$

where we clearly see an interplay between dispersive and extrinsic dissipative couplings. In our case, $g_{\kappa_e} \ll g_\omega$, and the main dissipative contributions to dynamical backaction come through crossed terms of the form $g_\omega g_{\kappa_e}$. Taking $\bar{\Delta} = \pm\Omega \gg \kappa$ we recover the results in the main text.

In the main text, we use Eq. S17 to fit the values of $\delta\Gamma$ extracted from our experiment. A taper-waveguide coupling efficiency calibration, along with the mechanical and optical spectra limit our fitting parameters to g_ω and g_{κ_e}.

S4. OPTOMECHANICALLY INDUCED TRANSPARENCY

In this section, we derive the equations for the dressed optical susceptibility due to the optomechanically induced transparency/absorption phenomena. We start with the important remark that in coherent spectroscopy the input laser is phase modulated, that is, one needs the substitution $\bar{\alpha}_{\mathrm{in}} \to \bar{\alpha}_{\mathrm{in}} e^{-i\phi_0\sin(\Omega_{\mathrm{mod}}t)}$ in Eq. S10. For weak modulations, $\phi_0 \ll 1$, we may approximate $\bar{\alpha}_{\mathrm{in}} e^{-i\phi_0\sin(\Omega_{\mathrm{mod}}t)} \approx \bar{\alpha}_{\mathrm{in}} + \frac{\phi_0\bar{\alpha}_{\mathrm{in}}}{2}e^{-i\Omega_{\mathrm{mod}}t} - \frac{\phi_0\bar{\alpha}_{\mathrm{in}}}{2}e^{i\Omega_{\mathrm{mod}}t}$, and the system is effectively driven by a strong pump tone, at frequency ω_l and two probes at $\omega_l \pm \Omega_{\mathrm{mod}}$.

The optical response to the phase-modulated input is modeled doing $a \to \bar{a} + a_+ e^{-i\Omega_{\mathrm{mod}}t} + a_- e^{i\Omega_{\mathrm{mod}}t}$. In order to simplify our discussion, we assume the pump laser is detuned several linewidths away from our optical resonance and $\Omega_{\mathrm{mod}} \gg \kappa$, in accordance with experimental conditions. In this case, the optical susceptibility acts as a bandpass filter, effectively filtering one of the sidebands of our probe tones. Assuming a red-detuned laser (the OMIT configuration), the phonon-creation process is suppressed and only a_+ is appreciable. Eq. S12 is rewritten as:

$$-i\Omega_{\mathrm{mod}}a_+ = i(\bar{\Delta}a_+ + g_\omega\bar{a}b_+) - \frac{\bar{\kappa}a_+ + g_{\kappa_e}\bar{a}b_+}{2} - \frac{g_{\kappa_e}b_+}{2\sqrt{\bar{\kappa}_e}}\bar{\alpha}_{\mathrm{in}} - \sqrt{\bar{\kappa}_e}\frac{\phi_0}{2}\bar{\alpha}_{\mathrm{in}}, \tag{S18}$$

where b_+ is the amplitude of the phononic field oscillating at Ω_{mod}. This quantity is obtained by solving the (classical) Langevin equation for b (obtained through the Hamiltonian of the system) with the substitution $b \to \bar{b} + b_+ e^{-i\Omega_{\mathrm{mod}}t} + b_- e^{i\Omega_{\mathrm{mod}}t}$.

The beating between the pump and probe tones will give rise to coherent mechanical oscillations, mediated by the optical force. In this classical picture, and under the same approximations above, Eq. S16 becomes

$$F x_{\mathrm{zpf}} = \hbar g_\omega \bar{a}^* a_+ e^{-i\Omega_{\mathrm{mod}} t} + i\frac{\hbar g_{\kappa_e}}{2\sqrt{\bar{\kappa}_e}}\left[\bar{\alpha}_{\mathrm{in}}\bar{a}^*\left(\frac{\phi_0}{2}e^{-i\Omega_{\mathrm{mod}} t} - \frac{\phi_0}{2}e^{i\Omega_{\mathrm{mod}} t}\right) - \left(\frac{\phi_0}{2}e^{-i\Omega_{\mathrm{mod}} t} - \frac{\phi_0}{2}e^{i\Omega_{\mathrm{mod}} t}\right)^* \bar{\alpha}_{\mathrm{in}}^*\bar{a}\right] - i\frac{\hbar g_{\kappa_e}}{2\sqrt{\bar{\kappa}_e}}\bar{\alpha}_{\mathrm{in}}^* a_+ e^{-i\Omega_{\mathrm{mod}} t}. \tag{S19}$$

In our system, the second term, which is rooted in the beating between the fluctuations (or oscillations) in the input and cavity fields is necessarily smaller than the other two. This is the case since it arises from the dissipative optomechanical coupling and scales with $\bar{a}$. For instance, since $g_{\kappa_e} \ll g_\omega$, the dispersive term (which also scales with $\bar{a}$) is obviously dominant over it. Furthermore, the last term, which is also dissipative in nature, scales with the photon flux in the waveguide, drastically increasing its importance relative to the second term. Joining all this information and writing the equation for b_+, we have

$$-i\Omega_{\mathrm{mod}} b_+ = -\left(i\Omega + \frac{\Gamma}{2}\right) b_+ + i g_\omega \bar{a}^* a_+ + \frac{g_{\kappa_e}}{2\sqrt{\bar{\kappa}_e}}\bar{\alpha}_{\mathrm{in}}^* a_+, \tag{S20}$$

which can be solved for b_+ in terms of a_+. Substituting this result back in Eq. S18 yields a dressed susceptibility for a_+, i.e.

$$a_+ = -\frac{\sqrt{\bar{\kappa}_e}\bar{\alpha}_{\mathrm{in}}\phi_0}{-2i\left(\bar{\Delta} + \Omega_{\mathrm{mod}}\right) + \bar{\kappa} - \frac{n_c\left[(\bar{\kappa}+2i\bar{\Delta})g_{\kappa_e} - 4i\bar{\kappa}_e g_\omega\right]\left[\left(2i\bar{\Delta}+2\bar{\kappa}_e-\bar{\kappa}\right)g_{\kappa_e} - 4i\bar{\kappa}_e g_\omega\right]}{4\bar{\kappa}_e^2(\Gamma - 2i\Omega_{\mathrm{mod}} + 2i\Omega)}}. \tag{S21}$$

Here, we see that the cavity response is modified by the optomechanical coupling. This is most relevant at $\bar{\Delta} = -\Omega_{\mathrm{mod}} = -\Omega$. In this case, and assuming a system well into the sideband-resolved regime, we have

$$a_+ \approx -\frac{\sqrt{\bar{\kappa}_e}\bar{\alpha}_{\mathrm{in}}\phi_0}{\bar{\kappa} + \frac{n_c(2\bar{\kappa}_e g_\omega + \Omega g_{\kappa_e})^2}{\Gamma\bar{\kappa}_e^2}}, \tag{S22}$$

which shows an effective enhancement in the cavity losses, or, equivalently a transparency window [7, 8]. This is equivalent to what has been verified for dispersive optomechanical systems, with the addition of the dissipative contribution. We notice that, in agreement with the discussion in the main text, the transparency window is favored by g_ω and g_{κ_e} with equal signs. This is also the case of backaction cooling under red-detuned excitation, as mentioned in the main text.

The generalization of the expressions above accounting for multiple, independent, mechanical modes (as is the case in our experiment) is done by simply adding extra optomechanical contributions to the optical response in the denominators of Eqs. S21 and S22, with modified mechanical linewidths, frequencies, and optomechanical couplings.

With this result, we are finally able to describe the scattering parameter $|S_{21}(\Omega_{\mathrm{mod}})|$ of the main text. This is done by using input-output relations, accounting for the phase-modulated laser. The outgoing field amplitude, α_{out} reads

$$\alpha_{\mathrm{out}} = \alpha_{\mathrm{in}} + \sqrt{\kappa_e}a + \frac{G_{\kappa_e}a}{2\sqrt{\kappa_e}}x, \tag{S23}$$

where the last term arises from the dissipative optomechanical coupling. In the linearized regime, this term is of little relevance, since it combines the intra-cavity field $\bar{a}$ and the dissipative coupling, which is small. Furthermore, it is not relevant for the modification of the cavity's susceptibility: it is generated by photons scattered from the cavity into the waveguide and therefore carries no information from the fluctuations in the cavity field. In that spirit, we neglect it in our analysis.

The outgoing field amplitude will have components at ω_l and $\omega_l \pm \Omega_{\mathrm{mod}}$, i.e. $\alpha_{\mathrm{out}} = \bar{\alpha}_{\mathrm{out}} + \alpha_{\mathrm{out},+}e^{-i\Omega_{\mathrm{mod}} t} + \alpha_{\mathrm{out},-}e^{i\Omega_{\mathrm{mod}} t}$. Combining this with the substitutions for the input and cavity fields, we have

$$\bar{\alpha}_{\mathrm{out}} = \bar{\alpha}_{\mathrm{in}} + \sqrt{\kappa_e}\bar{a} = \bar{\alpha}_{\mathrm{in}} r(\bar{\Delta}), \tag{S24}$$

$$\alpha_{\mathrm{out},+} = \frac{\phi_0\bar{\alpha}_{\mathrm{in}}}{2} + \sqrt{\kappa_e}a_+ = \frac{\phi_0\bar{\alpha}_{\mathrm{in}}}{2} r_{\mathrm{OM}}(\bar{\Delta} + \Omega_{\mathrm{mod}}), \tag{S25}$$

$$\alpha_{\mathrm{out},-} = -\frac{\phi_0\bar{\alpha}_{\mathrm{in}}}{2} + \sqrt{\kappa_e}a_- = -\frac{\phi_0\bar{\alpha}_{\mathrm{in}}}{2} r_{\mathrm{OM}}(\bar{\Delta} - \Omega_{\mathrm{mod}}), \tag{S26}$$

where we defined r and r_{OM} as the "bare" and dressed cavity reflectivities, respectively. Notice that for a red-detuned laser with $\bar{\Delta} = -\Omega$ and modulation frequency $\Omega_{\mathrm{mod}} = \Omega$, the on-resonance reflectivity is probed through $r_{\mathrm{OM}}(\bar{\Delta} + \Omega_{\mathrm{mod}})$, which is directly related to a_+. This is not the case for $r_{\mathrm{OM}}(\bar{\Delta} - \Omega_{\mathrm{mod}})$, which gives the response of the cavity two mechanical frequencies away from resonance, where the optomechanical contribution to the optical susceptibility is negligible. In fact, in this case, a reasonable approximation is $r_{\mathrm{OM}}(-2\Omega) \approx r(-2\Omega)$.

The scattering matrix element $|S_{21}(\Omega_{\mathrm{mod}})|$ is directly proportional to the output field amplitude at frequency Ω_{mod} [9]. This is given by

$$|S_{21}(\Omega_{\mathrm{mod}})| = \frac{\phi_0 |\bar{\alpha}_{\mathrm{in}}|^2}{2} \left| r(\bar{\Delta}) r^*_{\mathrm{OM}}(\bar{\Delta} - \Omega_{\mathrm{mod}}) - r^*(\bar{\Delta}) r_{\mathrm{OM}}(\bar{\Delta} + \Omega_{\mathrm{mod}}) \right|, \tag{S27}$$

where we omitted a scale factor. In our fittings throughout the text, an additional phase factor is introduced in order to handle any difference in the dispersion of the sidebands. This is done by making $r_{\mathrm{OM}}(\bar{\Delta}+\Omega_{\mathrm{mod}}) \to r_{\mathrm{OM}}(\bar{\Delta}+\Omega_{\mathrm{mod}}) e^{i\psi}$. Our fitting parameters are chosen to be $\bar{\Delta}$, ψ, and g_ω, with all the other parameters extracted from independent measurements shown in Figs. 2-4 in the main text.

S5. ANALYSIS FOR ACOUSTIC MODE 2

The results in Section S1 can be readily extended to the more realistic scenario in which each optical resonator is coupled to its own acoustic mode. Consider the system of coupled cavities in Fig. **1b** of the main text, where each optical resonator displays different resonance frequencies ω_i, $i = 1, 2$, and is coupled to independent acoustic modes with mechanical displacement x_i through a dispersive coupling G_{ω_i}. The generalization of the matrix $\mathbf{M}$ for this particular system is given by

$$\mathbf{M} = \begin{bmatrix} -i\left(\omega_1 - G_{\omega_1} x_1\right) - \frac{(\kappa_e + \kappa_i)}{2} & iJ \\ iJ & -i\left(\omega_2 - G_{\omega_2} x_2\right) - \frac{\kappa_i}{2} \end{bmatrix}. \tag{S28}$$

Once more, we are interested in the case where the frequencies of the bare optical resonators are degenerate, i.e. $\omega_2 = \omega_1$. Taylor expanding $\kappa_\mp$ and $\omega_\mp$ up to first order in the x_i, we obtain:

$$\begin{aligned} \kappa_\pm &= \kappa_i + \kappa_e \left(\frac{1}{2} \mp \frac{G_{\omega_1}}{4\sqrt{J^2 - (\kappa_e/4)^2}} x_1 \pm \frac{G_{\omega_2}}{4\sqrt{J^2 - (\kappa_e/4)^2}} x_2 \right), \\ \omega_\pm &= \omega_1 \pm 4\sqrt{J^2 - (\kappa_e/4)^2} - \frac{1}{2}\left(G_{\omega_1} x_1 + G_{\omega_2} x_2\right). \end{aligned} \tag{S29}$$

One can then define a dissipative (dispersive) coupling $G^{(i)}_{\kappa_{e\pm}}$ ($G^{(i)}_{\omega_\pm}$) associated to acoustic mode i:

$$G^{(i)}_{\kappa_{e\pm}} = \frac{d\kappa_\pm}{dx_i} = \begin{cases} \mp \frac{G_{\omega_i} \kappa_e}{4\sqrt{J^2 - (\kappa_e/4)^2}}, & i = 1, \\ \pm \frac{G_{\omega_i} \kappa_e}{4\sqrt{J^2 - (\kappa_e/4)^2}}, & i = 2, \end{cases} \tag{S30}$$

$$G^{(i)}_{\omega_\pm} = -\frac{d\omega_\pm}{dx_i} = \frac{G_{\omega_i}}{2}. \tag{S31}$$

In addition to recovering Eq. S5 in the $G_{\omega_2} \to 0$ limit, Eq. S31 also reveals that $G^{(1)}_{\kappa_{e\pm}} = -G^{(2)}_{\kappa_{e\pm}}$, meaning the sign of the dissipative coupling is flipped between different acoustic modes. This asymmetry between the behaviors of mechanical modes 1 and 2 is generated due to the fact that only one of the optical cavities is coupled to the waveguide. Furthermore, the dispersive coupling maintains its sign irrespective of the mechanical modes, $G^{(1)}_{\omega_\pm} = G^{(2)}_{\omega_\pm}$, implicating that optomechanical interactions dependent on the interplay between dispersive and dissipative coupling will display opposing behaviors between the different mechanical modes. In our experiment, acoustic modes 1 and 2 correspond to the independent mechanical breathing modes of the nanobeams. A frequency mismatch between them, due to fabrication fluctuations, enables their individual analysis of the impacts of the mechanically mediated waveguide-cavity interaction.

S6. NEARLY COLD-CAVITY TRANSDUCTION

Due to the high Q-factors and small modal volumes of our silicon devices, powers as low as 1 µW trigger nonlinear optical effects such as thermo-optical dispersion, two-photon absorption, and (consequently) free-carrier absorption

and dispersion [10, 11]. This is manifested in the optical spectra of our device, which is shown to be bistable in Fig. 3**b** of the main text.

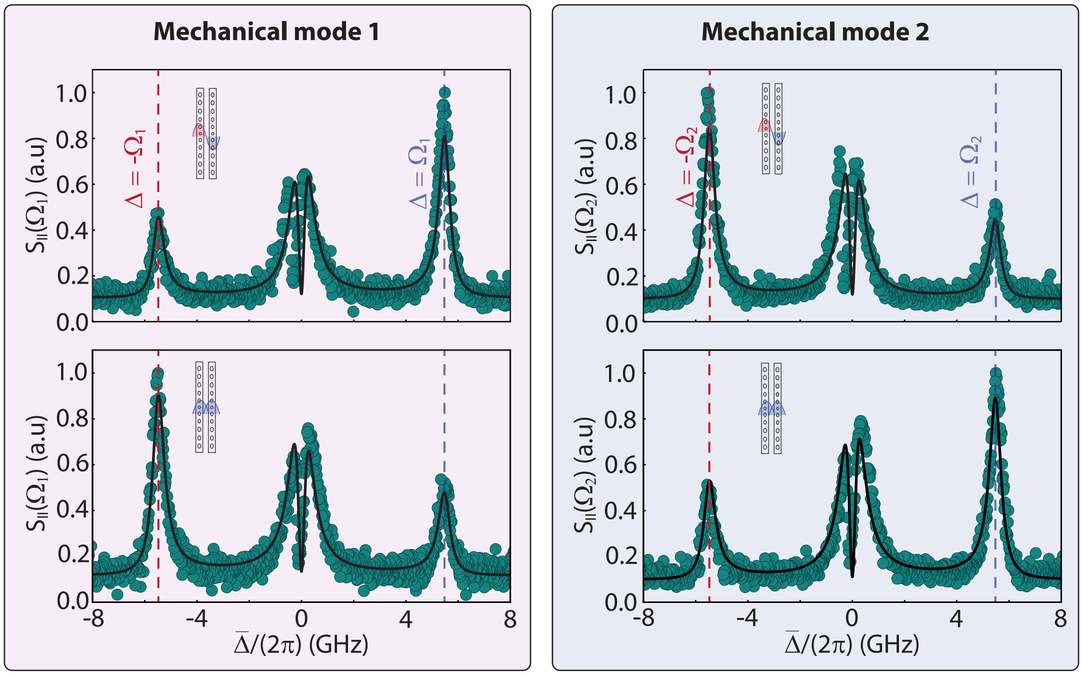

FIG. S1. Optomechanical transduction for mechanical modes 1 (left) and 2 (right), under excitation of the antisymmetric (top) and symmetric (bottom) optical modes.

In Fig. S1 we show the transduction spectra of mechanical modes 1 and 2 outside the optical bistable regime. We readily verify that the same asymmetries between red and blue-detunings is maintained for all combinations of optical and acoustic modes, in accordance with previously shown results. Furthermore, the black curves are the same theoretical estimates used before which were based on the values of dissipative and dispersive couplings extracted from backaction/OMIT experiments. The agreement between theory and experiment is poorer than for higher-power data. We attribute this to the low signal-to-noise ratio of the mechanical spectra for low optical powers and to a slight thermo-refractive shift of the resonance under near-resonant excitation.

S7. INDEPENDENT CONTROL OF DEGENERATE ACOUSTIC MODES

The interplay between dissipative and dispersive couplings allows for the augmentation or suppression of backaction-induced heating or cooling effects, depending on the relative sign between g_ω and g_{κ_e}, as demonstrated in the main text. For sufficiently high values of g_{κ_e}, a near-perfect cancellation of dynamical backaction can be achieved for a given optical/mechanical mode pair, enabling the independent control of virtually degenerate acoustic modes. This regime is obtained when the dissipative-to-dispersive coupling ratio is given by

$$\left|\frac{g_{\kappa_e}}{g_\omega}\right| = \frac{8\Omega_m^2 - \bar{\kappa}\bar{\kappa}_e}{\bar{\kappa}(\bar{\kappa} - 3\bar{\kappa}_e)\Omega_m + 4\Omega_m^3} \implies \left.\frac{\partial\delta\Gamma}{\partial g_{\kappa_e}}\right|_{\pm\Omega_m} = 0, \tag{S32}$$

as can be derived from Eq. S17. This is the point where variations on the mechanical linewidth are insensitive to an increase/decrease in the dissipative coupling and therefore constitute a minimum in $|\delta\Gamma|$ (given all other parameters are kept fixed).

Fig. S2 illustrates the theoretical optically-induced heating and cooling phenomena observed in mechanical modes 1 and 2 under both blue and red excitations. Remarkably, by considering optical and mechanical modes' properties akin to those outlined in the main text, a notable suppression of backaction effects can be realized at a critical ratio of $|g_{\kappa_e}/g_\omega| \approx 5.4\%$.

As an example, Fig. S2**a** demonstrates that when driving the differential optical mode at $\Delta = -\Omega_{1,2}$ one achieves enhanced cooling in acoustic mode 2 while strongly suppressing it in acoustic mode 1 at a critical ratio of $|g_{\kappa_e}/g_\omega| \approx$

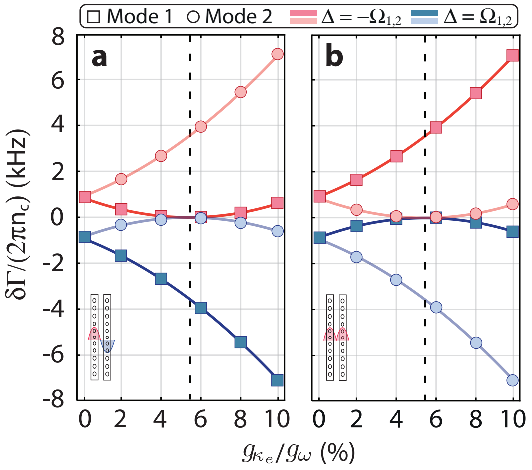

FIG. S2. **a** (**b**) Theoretical optically-induced modifications to the mechanical linewidth of mechanical modes 1 and 2 for the differential (common) optical modes scaled by the intracavity photon number, as a function of dissipative-to-dispersive optomechanical coupling ratio. Dashed line demarcates the threshold of near cancellation of either the backaction heating or cooling in a given optical/mechanical mode pair. Optical and mechanical parameters: $\Gamma_m/2\pi = 2.6\,\mathrm{MHz}$, $\Omega_{1,2}/2\pi = 5.5\,\mathrm{GHz}$, $\bar{\kappa}/2\pi = 550\,\mathrm{MHz}$, $\bar{\kappa}_e/2\pi = 150\,\mathrm{MHz}$, $g_\omega/2\pi = 350\,\mathrm{kHz}$.

5.4%. Repeating this analysis for $\Delta = \Omega_{1,2}$ (still considering the differential optical mode) signals a strong reduction of backaction heating in mode 2. As expected, opposite trends are showcased in Fig. S2**b** for the common optical mode. This decoupling between the responses of nearly degenerate acoustic modes suggests that for a given optical detuning it is possible to individually address and modify their states. This feature highlights one promising application in the field of quantum optomechanics, where information could be written and retrieved at will from specific mechanical modes, which could be therein used as versatile quantum memories [12] and/or building blocks for microwave-to-optical transducers [13].

Note. – Data, FEM and script files for generating each figure will be available at the *Zenodo* repository. [14].

SUPPLEMENTARY REFERENCES

[S1] H. Haus, *Waves and Fields in Optoelectronics* (Prentice-Hall, 1984).
[S2] Y. Yanay, J. C. Sankey, and A. A. Clerk, Physical Review A **93**, 063809 (2016).
[S3] F. Elste, S. M. Girvin, and A. A. Clerk, Physical Review Letters **102**, 207209 (2009).
[S4] T. Weiss, C. Bruder, and A. Nunnenkamp, New Journal of Physics **15**, 045017 (2013).
[S5] R. Kubo, Reports on Progress in Physics **29**, 255 (1966).
[S6] A. G. Primo, N. C. Carvalho, C. M. Kersul, N. C. Frateschi, G. S. Wiederhecker, and T. P. Alegre, Physical Review Letters **125**, 233601 (2020).
[S7] A. H. Safavi-Naeini, T. P. Alegre, J. Chan, M. Eichenfield, M. Winger, Q. Lin, J. T. Hill, D. E. Chang, and O. Painter, Nature 2011 472:7341 **472**, 69 (2011).
[S8] S. Weis, R. Rivière, S. Deléglise, E. Gavartin, O. Arcizet, A. Schliesser, and T. J. Kippenberg, Science **330**, 1520 (2010).
[S9] I. Shomroni, L. Qiu, D. Malz, A. Nunnenkamp, and T. J. Kippenberg, Nature Communications 2019 10:1 **10**, 1 (2019).
[S10] A. G. Primo, C. M. Kersul, R. Benevides, N. C. Carvalho, M. Ménard, N. C. Frateschi, P. L. De Assis, G. S. Wiederhecker, and T. P. Mayer Alegre, APL Photonics **6**, 086101 (2021).
[S11] P. E. Barclay, K. Srinivasan, and O. Painter, Optics Express, Vol. 13, Issue 3, pp. 801-820 **13**, 801 (2005).
[S12] A. Wallucks, I. Marinković, B. Hensen, R. Stockill, and S. Gröblacher, Nature Physics 2020 16:7 **16**, 772 (2020).
[S13] W. Jiang, F. M. Mayor, S. Malik, R. Van Laer, T. P. McKenna, R. N. Patel, J. D. Witmer, and A. H. Safavi-Naeini, Nature Physics (2023), 10.1038/s41567-023-02129-w.
[S14] A. G. Primo, P. V. Pinho, R. Benevides, S. Gröblacher, G. S. Wiederhecker, and T. P. M. Alegre, "Data and simulation files for: "Dissipative Optomechanics in High-Frequency Nanomechanical Resonators"," (2023).
